# Healthy collaborations are needed for mangrove land use and mosquito control

**DOI:** 10.2478/abm-2022-0013

**Published:** 2022-06-30

**Authors:** Asian Biomedicine

**Affiliations:** Faculty of Medicine, Chulalongkorn University, Bangkok 10330, Thailand

Mangroves are the dominant interface ecosystems between land and sea. They are breeding sites for mosquitoes and some distinctive shallow-water marine communities of bacteria and other organisms in tropical and subtropical waters. Urban development can modify the balance of existing ecosystems. In some instances, rapid industrialization and urban development can pollute mangrove forests with heavy metals, microplastic, and organic pollutants, contaminating sediments, plants, coral reefs, and mobile living organisms such as fish, and impairing biodiversity [1, 2, 3].

The modification of the complex ecosystem of mangrove forests has an impact on the microbial communities in mangrove sediments. The mangrove sediment-derived microorganisms may be a valuable reservoir of natural product diversity. The products could be utilized in the exploration of new antibiotics or drugs [4, 5] in addition to other benefits. Therefore, a decision to develop urban communities through encroachment of the mangrove ecosystem should be considered with care before it is implemented.

If, after a careful consideration, urbanization of mangroves becomes a desirable choice, strategies to control vector borne disease from mosquitoes are imperative. Good mosquito control strategies have implications for human health. Therefore, there is a need for a comprehensive planning for mosquito control and land use during urban development.

Comprehensive source reduction programs and larviciding are some of the control methods [6]. The long-term control by source reduction programs reduces vulnerability to mosquito issues such as population encroachment toward wetlands. Source reduction has been shown to be effective in minimizing vector borne disease [6]. However, integrating land use planning and mosquito control requires high level planning and resources. Ideally, higher government levels should take on this task, as they would be more effective in achieving the needed large-scale deployment of resources, which may be beyond the capacity of the local governments.

Another mosquito control strategy is through larviciding programs [6]. This strategy has been shown to be effective over the short-term, but the authorities involved may have to respond repeatedly, if or when subsequent problems arise. If governments allow residential development close to the mosquito habitats, the people in the area can develop vector borne diseases from mosquito transmissions. Further, some mangroves cover several administrative areas of the local governments. Without a good collaboration between these administrative entities that aims at effective mosquito control in all the mangrove areas, the prevention of effective vector borne diseases is difficult and can be worrisome to urban planning authorities. A healthy collaboration between stakeholders can assist in avoiding or resolving conflicts [6].

In this June 2022 issue of *Asian Biomedicine*, Maquart et al. [7] report the sampling of mosquito fauna, using BG-Sentinel (Biogents) and CDC-LT light traps (U.S. Centers for Disease Control) in the mangrove forest of Koh Kong province, Cambodia, to understand their mosquito diversity, and subsequently analyze their virome. The information will be useful if an encroachment of the mangrove is planned in the future. They found 12 medically important species. Two of the species, *Aedes* (*Stegomyia*) *albopictus* and *Culex vishnui*, can be found throughout the year. They have important implications for arboviral diseases in humans and animals caused by mosquito vector borne transmission of infectious agents, such as Zika, dengue, or chikungunya viruses. If new arboviruses are found in this area, these species would have the potential to be a bridge between the various arboviruses and humans or animals.

Mangrove ecosystems are beneficial to humans. Caution is required in planning urban use of land by encroaching on the ecosystem. Effective mosquito control strategy should be integrated with land use planning through a healthy collaboration between parties involved.
